# Blood Ammonia as a Possible Etiological Agent for Alzheimer’s Disease

**DOI:** 10.3390/nu10050564

**Published:** 2018-05-04

**Authors:** Yan Yan Jin, Parul Singh, Hea-Jong Chung, Seong-Tschool Hong

**Affiliations:** Department of Biomedical Sciences and Institute for Medical Science, Chonbuk National University Medical School, Jeonju, Chonbuk 54907, Korea; kimyanyan@jbnu.ac.kr (Y.Y.J.); parulsinghmedicalschool@gmail.com (P.S.); hjchung@jbnu.ac.kr (H.-J.C.)

**Keywords:** Alzheimer’s disease, Mediterranean diet, blood ammonia level, Lactobacillus, gut microbiota

## Abstract

Alzheimer’s disease (AD), characterized by cognitive decline and devastating neurodegeneration, is the most common age-related dementia. Since AD is a typical example of a complex disease that is affected by various genetic and environmental factors, various factors could be involved in preventing and/or treating AD. Extracellular accumulation of beta-amyloid peptide (Aβ) and intracellular accumulation of tau undeniably play essential roles in the etiology of AD. However, interestingly enough, medications targeting Aβ or tau all failed and the only clinically efficient medications for AD are drugs targeting the cholinergic pathway. Also, a very intriguing discovery in AD is that the Mediterranean diet (MeDi), containing an unusually large quantity of Lactobacilli, is very effective in preventing AD. Based on recently emerging findings, it is our opinion that the reduction of blood ammonia levels by Lactobacilli in MeDi is the therapeutic agent of MeDi for AD. The recent evidence of Lactobacilli lowering blood ammonia level not only provides a link between AD and MeDi but also provides a foundation of pharmabiotics for hyperammonemia as well as various neurological diseases.

## 1. Introduction

As the population ages, the incidence and prevalence of age-related diseases have been increasing year by year, posing a new global dilemma. Alzheimer’s disease (AD) is one of the most common age-related diseases and is a serious public health challenge, with over 5 million new cases each year, over 33 million patients worldwide and an economic burden exceeding half a trillion USD [1]. It has been reported that the number of patients with AD is predicted to be 52.9 million by 2030 and 94.9 million by 2050, which is almost triple the existing population affected [1].

Characterized by dementia, AD starts from short-term memory loss, progresses into cognitive deficits and ultimately leads to death [2]. Patients increasingly rely on others for assistance, while the pressures of costly and prolonged treatments for AD patients involve psychological, physical, economic and social elements. The cause and mechanism of AD are still poorly understood so that several hypotheses are competing against each other to explain the etiological mechanism of AD. In the last decade, AD researches have focused on amyloid plaques, neurofibrillary tangles and the cholinergic pathway in the progression of AD. In accordance with the trends of AD researches, there has been an investigation with a large number of therapeutic agents such as anti-Aβ regimens, β-site peptide cleaving enzyme-1 (BACE1) inhibitors, *N*-methyl-d-aspartate antagonists and cholinesterase inhibitors to modulate those pathways [3]. However, those efforts to develop therapeutic agents for AD have not been successful in spite of serious scientific effort and still remains a most intractable scientific challenge [4,5].

The failure in developing efficient therapeutic agents for AD might originate from the fact that AD is a typical example of complex diseases. It has been well known that a combination of various genetic, environmental and lifestyle factors cause AD. Among the various determinant factors for AD, the significance of blood ammonia as an etiological agent has not been widely noted. Ammonia, which is produced as an end-product of protein catabolism, has been devoted much attention for its strong neurotoxicity. Although acute hyperammonemia manifests as encephalopathy, the clinical manifestations of chronic, slightly elevated blood ammonia levels have not been studied. However, considering the neurotoxic nature of ammonia, it would be reasonable to speculate that chronically elevated levels of ammonia are associated with AD. In fact, several initial studies declared the association between AD and blood ammonia level [6,7], providing a possible link between ammonia level and AD.

The multiple different facets of AD and related pathophysiology obviously require us to consider a new paradigm to develop therapeutic agents for AD. Targeting gut microbiota could be an example of a new paradigmatic approach for the development of therapeutic agents for AD. Recent evidence show that gut microbiota play an intricate role in many different neuronal functions as well as various neurological diseases such as Autism spectrum disorder (ASD), Parkinson’s disease (PD), multiple sclerosis (MS), mood disorders, Gulf War Illness (GWI), multiple sclerosis, neuromyelitis optica spectrum disorders, Huntington disease and amyotrophic lateral sclerosis and so on [8,9,10,11,12,13,14]. Considering the fact that the gut-brain axis is the most significant signaling between the gastrointestinal (GI) tract and the central nervous system (CNS) in the body, it would not be surprising to note that gut microbiota act as important etiological agents for neurological diseases or modulators for neuronal function. In this context, recent findings of some Lactobacillus strains reducing blood ammonia levels throw a light on how gut microbiota affects neurological diseases and neuronal functions. Because blood ammonia is a waste metabolite acting as the strongest neurotoxin in the body, chronically elevated ammonia levels could lead to neurodegeneration which can manifest as AD. Therefore, the fact that certain Lactobacilli strains efficiently reduce blood ammonia levels makes us ponder whether the characteristic of a Mediterranean diet (MeDi), preventing AD roots on the consumption of unusually large quantity of Lactobacilli.

## 2. The Etiological Mechanism of AD

AD is a multifactorial neurodegenerative disease in which environmental risk factors (i.e., toxins, xenobiotics, ultraviolet radiation and pollutants) and genetic factors (including mutations of amyloid precursor protein (APP), presenilin genes (PSEN1 and 2) and allelic variation in apolipoprotein E (Apo E)) interact to enhance the incidence [15,16]. Despite world-wide efforts to elucidate the etiology of AD, progress towards this goal is surprisingly limited. The etiological mechanism for most cases of AD is still mostly unknown. But many hypotheses have been proposed to explain the AD’s causes. These include the Amyloid hypothesis, the Tau hypothesis, Genetics, the Cholinergic hypothesis and other hypotheses.

**Figure 1:**
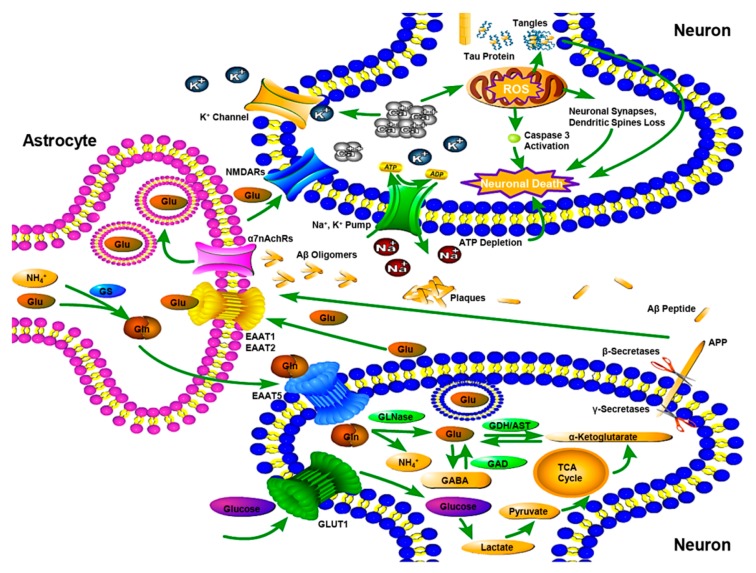
Mechanism of Amyloid hypothesis, Tau hypothesis and Glutamine-Glutamate cycle in an Alzheimer’s disease (AD) brain. In the presynaptic neuron, mutational amyloid precursor protein (APP) located in cell membrane is cleaved by β- and γ-secretases, produces excess Aβ peptides, which promote production of Aβ oligomers. Excess neuronal Aβ oligomers flow onto surrounding astrocytes to bind with α7nAChRs. The signals from α7nAChRs result in astrocytes’ glutamate exocytose to activate the extrasynaptic *N*-methyl-d-aspartate receptors (NMDARs) of the astrocytes’ partner neurons. The resulted signals evoke Ca^2+^ surges and induce dysfunctional mitochondria with pumping out reactive oxygen species (ROS), which trigger a tau hyperphosphorylation, oxidative damage, caspase 3 activation, excess production of ROS, thereby severing communications within the astrocytes’ neurons and beyond and destroying neuronal synapses, dendritic spines. Consequently Ca^2+^ surges increase in extracellular K^+^, which induces adenosine triphosphate (ATP) depletion, cell swelling and cell death. Abnormal phosphorylation of tau results in the transformation of normal adult tau into neurofibrillary tangles (NFTs) and disruption of the axonal form of microtubules. Glutamate released from presynaptic terminals transported via the excitatory amino acid transporter (EAAT) 1, 2 into astrocytes, where is transformed to glutamine by the glutamine synthetase (GS). Subsequently, the glutamine taken up by neurons is converted to glutamate and the synthesis of α-ketoglutarate and glutamate occurs, after which is then metabolized into Gamma-aminobutyric acid (GABA) by glutamate decarboxylase (GAD). AST, aspartate transaminase; EAAT 5, excitatory amino acid transporter 5; GDH, glutamate dehydrogenase; GLN, glutamine; GLNase, glutaminase; GLU, glutamate.

The most commonly used hypothesis is the amyloid hypothesis and recent studies have also focused on the role of Aβ oligomers in synaptic impairment [17,18,19], which accumulate to form extracellular fibrillar amyloid plaques, one of the pathological features in AD [20]. As a transmembrane protein, APP penetrates through the cell membrane. It is critical to neuron growth, post-injury repair and survival [21]. The normal APP is processed by α-secretase, while mutational APP is cleaved by β- and γ-secretases, altering the balance between the production and clearance of Aβ peptide [22]. Both the activated neurons and the associated astrocytes produce Aβ42 oligomers [19]. When excess neuronal Aβ42 oligomers start flowing out of the neurons onto their surrounding astrocytes, besides aggregating or diffusing in the extracellular periphery, neurons and astrocytes are permitted with Aβ42 oligomer-binding receptors [19]. Because of the functional and intimate physical interdigitations in the neurons’ client group, the neuron-associated astrocytes’ α7nicotinic Ach receptors (α7nAChRs) are directly bound to the Aβ42 oligomers [23]. The signals from α7nAChRs result in glutamate being exocytosed by the astrocytes, which they have been taking up from the neuronal synapses [23]. The discharged glutamate activates the extra synaptic *N*-methyl-d-aspartate receptors (NMDARs) of the astrocytes’ partner neurons [23,24]. The resulting signals evoke Ca^2+^ surges to induce dysfunctional mitochondria to pump out reactive oxygen species (ROS), which trigger tau hyperphosphorylation, oxidative damage, caspase 3 activation, excess production of ROS, nitric oxide (NO) and vascular endothelial growth factor (VEGF), thereby severing communications within the astrocytes’ neurons and beyond destroying neuronal synapses, dendritic spines [23,25]. Consequently, Ca^2+^ surges increase the concentration of extracellular K^+^, inducing adenosine triphosphate (ATP) depletion, cell swelling and cell death [26] (Figure 1).

Tau is a microtubule-associated protein synthesized by neuronal cells. It is responsible for stabilization of the microtubules to support neuronal functions such as cell growth, axonal morphology and polarity which becomes missorted in AD [27]. It is considered that excess Aβ42 oligomers production promotes tau hyperphosphorylation to evoke the disease cascade [28]. Threads of tau proteins accumulate to eventually form intracellular neurofibrillary tangles, one of pathological features in AD [29]. Subsequently, the axonal form of microtubules is destroyed, which is the neuronal transport system, while the structure of neuronal cytoskeleton is disintegrated [30]. These variations result in a cascade of events, including malfunctions in neuronal biochemical communication and later cell deaths [29]. While the AD’s pathological features, such as amyloid plaques and neurofibrillary tangles, are recognized, the etiological mechanisms have not been clearly. Thus, this lesser understanding about the onset of AD could be the possible reason behind the lack of an effective cure of the disease to date (Figure 1).

## 3. Association of AD with Low Degree of Chronic Hyperammonemia

Since the first description of AD in 1907, a great number of possible etiologic factors have been advanced. Among these factors, ammonia deserves special interest for its demonstrated toxicity on CNS function. Depending upon the duration and magnitude of exposure, elevated brain ammonia results in typical neurological symptom common to AD, including memory dysfunctions, cognitive and spatial learning; and pathophysiological aberrations include the following: mitochondrial dysfunction, disruption of cellular glucose metabolism, neurotransmission and regulation of inflammatory responses.

Ammonia is a normal end product of human tissues’ metabolism. Nevertheless, it is a highly neurotoxic compound at even sub-millimolar concentrations [26,31]. Thus, ammonia detoxification in organisms is indispensable. In the brain, in either normal or hyperammonemic conditions, the route of ATP-dependent formation of glutamine by glutamine synthetase (l-glutamate:ammonia ligase (ADP-forming; E.C.6.3.1.2)) (GS) is predominantly used for ammonia removal [32]. And, astrocyte is the exclusive to modulate GS expression for the limitation of brain ammonia rather than neuron [33].

**Figure 2:**
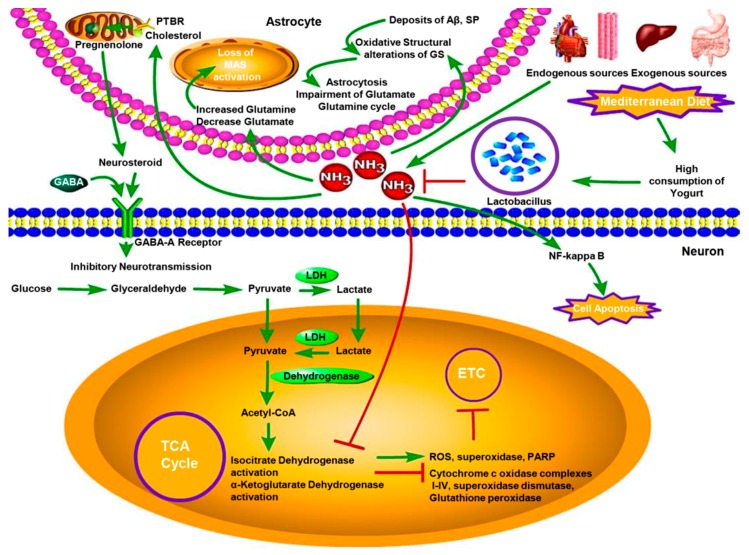
The neurotoxicity of hyperammonemia and protective effect of Lactobacillus in the AD brain. Blood ammonia from endogenous sources and exogenous sources crosses the blood-brain barrier and builds up in the brain regulating into increasing of glutamine, while decreasing of glutamate, which leads to the loss of malate-asparate shuttle (MAS) activity to induce the reduction of pyruvate/lactate ratio. Unrelated to MAS activity, high ammonia concentration suppresses the activities of alpha-ketoglutarate dehydrogenase and isocitiate dehydrogenase to increase the activity of ROS, superoxidase and Poly ADP-Ribose polymerase (PARP) in AD brain mitochondria. That can also decrease the activity of cytochrome c oxidase, complexes I–IV, superoxidase dismutase and glutathione peroxidase to inhibit mitochondrial electron transport chain (ETC). Hyperammonemia stimulates the PTBR localised uniquely on the astrocytic outer mitochondrial membrane inducing increased pregnenolone and cholesterol transport. The former is the precursor of “neurosteroids,” which have positive allosteric modulatory properties on the neuronal GABA-A receptor leading to increased inhibitory neurotransmission. In AD brain, the density of extracellular deposits of Aβ and SP in the cortex induces the oxidative structural alterations of GS by interacting with it, as well as enhances the neurotoxicity of Aβ. That causes loss of astrocytic GS activity and induces the impairment of Glutamate-Glutamine cycle and astrocytosis. Lactobacillus stains supresses the neurotoxicity of hyperammonemia in AD brain by reducing blood ammonia levels. LDH, lactate dehydrogenase.

In the AD brain, the density of extracellular deposits of Aβ and senile plaques (SP) in the cortex induces the oxidative structural alterations of GS by interacting with it as well as enhances the neurotoxicity of Aβ [34]. This causes loss of astrocytic GS activity and induces the impairment of Glutamate-Glutamine cycle and astrocytosis [35]. Thus, it is a general phenomenon that brain ammonia concentration increases by lower brain GS activity with age. A previous study has shown that the ammonia levels in AD group were prominently higher than in control group [36], while others have proved that there was a significant difference between post-prandial blood ammonia levels and the values before meals. The patients with high blood ammonia levels showed tri-phasic waves, the kind of electroencephalographic (EGG) which is suggestive of hepatic encephalopathy (HE) [6]. And all patients in these studies were free of other diseases and exogenous ammonia sources. These results support amyloid plaque formation and abnormal ammonia metabolism in AD brain (Figure 2).

Ammonia significantly disrupts mitochondrial function and cerebral energy metabolism. High ammonia concentration suppresses the activities of alpha-ketoglutarate dehydrogenase and isocitrate dehydrogenase to increase the activity of ROS, superoxidase and Poly ADP-Ribose polymerase (PARP) in AD brain mitochondria [37]. This can also decrease the activity of cytochrome c oxidase, complexes I–IV, superoxidase dismutase and glutathione peroxidase to inhibit mitochondrial electron transport chain (ETC) [38,39]. Consequentially, the ammonia-induced stress condition contributes to the changes in mitochondria morphology, the regulation of the balance between mitochondrial fission and fusion and a reduced rate of mitochondrial axonal transport [40,41] (Figure 2).

Besides mitochondrial dysfunction, energy metabolism is affected in AD brain. The reduction in glycolytic process was observed by comparing the enzymatic activity of glucose transporters, pyruvate dehydrogenase (PDH) [42], hexokinase and enzymes of the tricarboxylic acid (TCA) cycle between AD group and control group [43]. In addition, ammonia-induced inhibition of the TCA cycle in brain was observed along with the demonstration of how the increased astrocytic glutamine reduces glutamate concentration, leading to the loss of malate-asparate shuttle (MAS) activity to result in the reduction of pyruvate/lactate ratio. Unrelated to MAS activity, hyperammonemia inhibits PDH via regulating decarboxylation of alpha-ketoglutarate [44] (Figure 2).

Gamma-aminobutyric acid (GABA) is the chief inhibitory neurotransmitter in the CNS to play a critical role in AD, being extensively studied in AD and hyperammonemia. Some papers have proven that elevated ammonia level increase the GABA release to enhance the GABAergic system in AD which upregulates inhibitory neurotransmission to cause cognitive deficits in AD [45]. Further, one theory has been postulated to explain that hyperammonemia stimulates the“peripheral-type” benzodiazepine receptor (PTBR) [46]. PTBR is localized uniquely on the astrocytic outer-mitochondrial membrane and induces increased pregnenolone and cholesterol transport. The former is the precursor of “neurosteroids” [47], which have positive allosteric modulatory properties on the neuronal GABA-A receptor leading to increased inhibitory neurotransmission [48]. It is generally thought that neuroinflammation is a pathological feature of AD. However, some findings indicate that high ammonia concentration leads to the neuronal cell apoptosis via nuclear factor-kappa B (NF-κB) [49], which plays an indispensable role to modulate inflammatory responses, impairment of mitochondrial function, immunity and apoptosis via regulating the activity of NO, superoxide, phospholipase A2 (PLA2), peroxynitrite and inducible nitric oxide synthase (iNOS), NADPH oxidase (NOX) and mitochondrial dysfunction [50,51]. Even there are some evidences to support the association among high ammonia concentration, GABA and neuroinflammation but the mechanisms and pathways are still not clear (Figure 2).

These observations support that ammonia is a potential neurotoxic factor which induces pathophysiological progression and typical symptoms of AD. Speculatively, the aim to predict the way of maintaining a normal ammonia level in brain might be the predominant therapeutic of AD.

## 4. Effect of Lactobacilli on Lowering Blood Ammonia Levels

High blood ammonia is an excessive accumulation of ammonia in the blood. It occurs due to increased production or decreased clearance of ammonia [32]. In general, ammonia is produced from both exogenous and endogenous sources [52]. Endogenous sources involve hydrolysis of proteins, degradation of amino acids, deamination of amino-purines and oxidative deamination of primary amines while exogenous sources generates substantial amount of ammonia in the gut due to bacterial degradation of urea and deamination of amino acids [53]. Existing evidences indicate that ammonia accumulation in the brain damages neurons and can lead to various neurological abnormalities. Low levels of ammonia (50–250 μM) are not toxic to the brain due to protection from the blood-brain barrier but high blood ammonia levels (1–10 mM) can cross the blood-brain barrier [54] and build up in the brain, resulting into neurological disorders such as AD. Ammonia levels within 50–250 μM concentration is considered to be in a tolerable range to ensure the systemic functions. Concentrations exceeding 1 mM are usually toxic. Ammonia exposure at 1–10 mM produced swelling of immunochemically-identified astrocytes and at 10 mM resulted in macroscopic tissue swelling, with slice thickness increasing by about 30% among the various bacteria, lactic acid bacteria are thought to have beneficial effects to the host through various mechanisms such as anti-tumorigenic, anti-inflammatory and pathogen-exclusion properties [55]. Increasing evidences indicate that Lactobacilli have been associated with promoting the growth of non-urease producing species, lowering the intestinal pH and reducing the absorption of ammonia in the colonic lumen [56]. Study shows that oral and rectal administration of *Lactobacillus plantarum* decreased both blood and fecal ammonia levels in rodent models of hyperammonemia [57]. A slight improvement in alanine aminotransferase (ALT) serum levels and in liver histopathology was shown after administration of Lactobacilli in a rat model of mild HE induced by thioacetamide (TAA) [58]. In this study, a new strain of *Lactobacillus plantarum* was constructed which overproduced alanine dehydrogenase and consumed in vitro higher amounts of ammonia than its wild-type counterpart. When given at doses of 10^9^ CFU in vivo to mice suffering from acute liver failure with hyperammonemia, this modified strain had the same ability to decrease blood ammonia, to decrease mortality and to consume gut ammonia, such as that of wild-type *Lactobacillus plantarum* [59]. Some clinical studies performed in patients with liver diseases suggest that *Lactobacillus* strains were able to decrease blood ammonia level and improve neuropsychological symptoms (Table 1). It appears that the reduction in blood ammonia level occurred due to reduced bacterial urease activity and the alleviation of potential of hydrogen (pH) which helps to absorb ammonia in the gut [60].

A key question is whether the effect of *Lactobacillus* found in yogurt on lowering blood ammonia levels has a significant impact on the pathology of AD. The first hint to the answer came from a recent study where the efficacy of *Lactobacillus helveticus* NS8 was tested in preventing cognitive decline and anxiety-like behavior in hyperammonemia rats [61]. Chronic hyperammonemia was induced in rats by intraperitoneal injection of ammonium acetate. And, hyperammonemia rats were then given *Lactobacillus helveticus* NS8 (10^9^ CFU mL^−1^) in drinking water as a daily supplementation. Although this study didn’t measure the difference in blood ammonia levels, it still presents interesting results that the administration of *Lactobacillus helveticus* NS8 significantly reduced the level of inflammatory markers, decreased 5-HT metabolism and restored cognitive function. Another recent study showed that the probiotic (containing *Lactobacillus acidophilus, Lactobacillus casei, Bifidobacterium bifidum* and *Lactobacillus fermentum*) administration for three months has favorable effects on Mini–Mental State Examination (MMSE) score, malonyl dialdehyde (MDA), high sensitive C-reactive protein (hs-CRP), markers of insulin metabolism and triglycerides levels of the AD patients [62]. In this study also, author didn’t notice the importance of blood ammonia level. We strongly believe that it is important to check blood ammonia level considering the neurotoxicity of ammonia in the context of AD.

## 5. Consumption of a Large Quantity of Lactobacilli in the Medi as a Reason for a Lowered Incidence Rate of AD in Mediterranean

The dietary pattern of populations bordering the Mediterranean Sea is called Medi and has been widely reported for its model of healthy eating and is often linked to the lower incidence rate of AD in the Mediterranean region [67]. Population-based cross-sectional cohort study conducted in Greece [68] and The Three-City Study conducted in France [69] showed that the adherence to a Medi was associated with better global cognitive function. Another prospective longitudinal cohort study was conducted in Bordeaux, France which examined 1410 non-demented elderly individuals [70]. They were re-examined at least once over 5 years. Some neuropsychological tests such as MMSE, Isaacs Set Test, Benton Visual Retention Test and Free and cued Selective Reminding Test were used to evaluate the cognitive ability. The major finding of this study was that the higher adherence to the Medi showed small number of errors in MMSE and slower decline of cognitive function [71].

Typical components of Medi include proportionally high consumption of dairy products (mostly as yogurt and cheese), high intake of vegetables, legumes, fruits and cereals, high intake of unsaturated fatty acids, (mostly in the form of olive oil) and a low intake of meat and poultry [72]. Medi effect on cognitive health is mediated by several biological mechanisms such as reduced risk of coronary heart disease, diabetes, dyslipidemia, hypertension and metabolic syndrome [73]. These conditions are also related to AD. Higher adherence to Medi has been related to improved glucose metabolism and insulin sensitivity, promoting metabolic control. In recent years, dietary patterns in relation to diseases have been in focus [74]. This useful approach acknowledges the fact that food and nutrients act in concert and are biologically interactive. Lately, there is a growing interest for Medi because of its impact on overall mortality [75].

Medi has been the most investigated dietary pattern in many epidemiological studies. Several studies on Medi such as the Memory and Aging Project (MAP) [76], Washington Heights–Inwood Columbia Aging Project (WHICAP) [77], Reasons for Geographic and Racial Differences in Stroke (REGARDS), ref. [78] and the Chicago Health and Aging Project (CHAP) [79] have found clear protective effects against cognitive decline. Inflammation has been found to be associated with a higher risk for AD. CRP is a non-specific inflammatory marker that has been detected in neuritis plaques and neurofibrillary tangles in the brains of patients with AD [80]. Observational and interventional studies have showed that higher adherence to the Medi has been associated with lower CRP levels [81]. In the ATTICA epidemiological study, participants on the highest tertile of the MeDi score had 20% lower CRP levels and in the Nurses’ Health Study, there was a 24% reduction in CRP levels for subjects belonging to the upper quintile of a MeDi adherence index [82]. Many studies implicate oxidative damage in the pathogenesis of AD with neurons at risk having increased lipid peroxidation, nitration, free carbonyls and nucleic acid oxidation. There is evidence that decreased oxidative stress was found in people who adhere to MeDi. It may have increased plasma brain-derived neurotrophic concentration [83]. This can explain the association of MeDi with lower risk of AD.

Regarding causative factors in AD, various neurotoxins have been proposed but the effect of ammonia as a potent neurotoxin in relation to the pathology of AD has received less attention [84]. A high concentration of ammonia causes deleterious effects on the cell. Several studies indicate that upon ammonia-induced stress condition activity of PARP increased in brain mitochondria. Some studies have reported detection of elevated blood ammonia levels in the patients with AD [85]. Others have found that ammonia concentrations of lumen and dialysate samples were lower in MeDi group than Scandinavian diet group, as well as, the group of whole components of MeDi showed lower ammonia level than cereal group or fruit and vegetable group [86]. It is important to notice that water-rich dairy product (yogurt) is one of the main characteristics of MeDi [87]. Adherence to MeDi could allow lactic acid bacteria in the gut through the competitive inhibition due to high consumption of yogurt [88]. In addition, existing evidence suggests that administration of Lactobacilli, contained in yogurt, helps in reducing elevated blood ammonia levels [89]. Lactobacilli reduce the blood ammonia level by stabilizing physiological luminal permeability. On the other hand, Lactobacilli also promote the growth of non-urease producing microflora bacteria followed by depressing ammonia production and release into the portal system [90]. Therefore, higher adherence to the MeDi may be able to remove neurotoxin effects of ammonia in the brain by lowering the blood ammonia levels and this could, at least partially, explain the lowered incidence rate of AD in Mediterranean.

## 6. Discussion

Various epidemiological studies have consistently shown that the MeDi slow cognitive decline in older adults reduces the risk of mild cognitive impairment, a transitional stage between the cognitive decline of normal aging and most interestingly, lowers the risk of AD. Despite the obvious fact that the MeDi is beneficial for healthy brain, the scientific reason is unclear even now. The MeDi consists of especially large quantity of yogurt, which means that Mediterraneans consume an extraordinary quantity of Lactobacilli. In this context, recent evidence showing that some *Lactobacillus* strains efficiently reduced blood ammonia levels cast a light on a historically long, nagging question on why the MeDi is beneficial for a healthy brain. We believe that Lactobacilli, especially abundant in the MeDi, help to maintain a low level of blood ammonia level. Because ammonia is the strongest natural neurotoxin, it would be unrealistic that maintaining blood ammonia at low levels does not contribute positively for health of brain.

The recent evidence of Lactobacilli lowering blood ammonia level not only provides a link between AD and MeDi but also suggest a possible role of gut microbiota in neurological diseases and neuronal functions. Because there could be a plenty of intestinal bacteria among the constituent of gut microbiota lowering blood ammonia levels, investigating various strains of intestinal bacteria will provide a foundation for pharmabiotics for hyperammonemia as well as various neurological diseases.

## Figures and Tables

**Table 1:** Limited clinical trials in patient with Liver disease.

Method	Subjects	Treatment	Duration of Treatment	Result	References
RCT	40	*Enterococcus faecium* SF68 or lactulose	Three periods of 4 weeks with 2 weeks of drug-free intervals	Reduction in blood ammonia levels, improved neurocoginitive (Reitan’s) test	[63]
RCT	55	synbiotic preparation (*n* = 20), fermentable fiber alone (*n* = 20), or placebo (*n* = 15)	30 days	Increment in the fecal content of non-urease-producing *Lactobacillus* species, significant reduction in blood ammonia levels and reversal of minimal hepatic encephalopathy (MHE) in 50% of patients	[64]
RCT	160	lactulose, probiotics and L-ornithine L-aspartate (LOLA)	3 months	Reduction in blood ammonia levels, significantly improved MHE	[65]
RCT	25	probiotic yogurt	2 months	significant rate of MHE reversal	[66]

RCT: randomized clinical trial.
